# Elephants born in the high stress season have faster reproductive ageing

**DOI:** 10.1038/srep13946

**Published:** 2015-09-14

**Authors:** Hannah S. Mumby, Khyne U. Mar, Adam D. Hayward, Win Htut, Ye Htut-Aung, Virpi Lummaa

**Affiliations:** 1 Department of Animal and Plant Sciences, University of Sheffield, Western Bank, Sheffield S10 2TN, United Kingdom; 2 Institute of Evolutionary Biology, University of Edinburgh, Edinburgh EH9 3JT, United Kingdom; 3 Ministry of Environmental Conservation and Forestry, Myanma Timber Enterprise, Yangon, Myanmar; 4 Department of Veterinary Medicine, Yezin University, Myanmar

## Abstract

Senescent declines in reproduction and survival are found across the tree of life, but little is known of the factors causing individual variation in reproductive ageing rates. One contributor may be variation in early developmental conditions, but only a few studies quantify the effects of early environment on reproductive ageing and none concern comparably long-lived species to humans. We determine the effects of ‘stressful’ birth conditions on lifetime reproduction in a large semi-captive population of Asian elephants (*Elephas maximus*). We categorise birth month into stressful vs. not-stressful periods based on longitudinal measures of glucocorticoid metabolites in reproductive-aged females, which peak during heavy workload and the start of the monsoon in June-August. Females born in these months exhibit faster reproductive senescence in adulthood and have significantly reduced lifetime reproductive success than their counterparts born at other times of year. Improving developmental conditions could therefore delay reproductive ageing in species as long-lived as humans.

Whilst senescent declines in reproduction as well as survival are now evident across taxa^1^, far less is currently known of the factors allowing some individuals to better cope with growing old than others. One contributor to such differences in reproductive ageing may be variation in early developmental conditions. This is feasible because it is known that early life conditions affect a series of other later life characteristics including metabolic traits^2^ into adulthood, causing differences in survival, health and reproduction^3^,^4^. In line with this, some evidence now links variation in early life conditions to differences in measures of survival and reproductive senescence in some invertebrates^5^,^6^,^7^,^8^,^9^ and short-lived birds^10^. In contrast, only very few studies on mammals or long-lived birds have investigated the effects of early developmental conditions on variation in later rates of reproductive senescence. In red deer (*Cervus elaphus*)^11^, tawny owls (*Strix aluco*)^12^ and red squirrels (*Tamiasciusus hudsonicus*)^13^, individuals born in poor conditions (as measured by high natal density, high prey density and low food abundance respectively) had higher rates of subsequent reproductive senescence than their counterparts born in better conditions. Under energetic constraints models, these patterns could represent the long-term consequences of high energetic demands placed on the mother or offspring under poor early life conditions^14^. Even red deer only live for at maximum around 15 years, raising the questions of how applicable such findings might be for species that reproduce not just in a single breeding season, but throughout the year and over several decades rather than years, such as humans. Moreover, as population density or food abundance are often correlated across years, poor birth year conditions may also reflect less favourable subsequent conditions, and there is therefore a need for studies that are able to contrast cohorts with differing early developmental conditions, but similar later environments.

The one long-lived species studied in detail are humans. Extensive evidence from humans shows that conditions experienced during early development have profound effects on metabolism and later-life health as well as reproductive outcomes such as age at menopause. For example low birth weight is related to a wide range of outcomes including higher risk of diabetes^15^, cancer^16^ and cardiovascular disease^17^ in adulthood, and birth during a period of food shortage can be related to the size of own offspring^18^ and ability to cope with poor food conditions in later life^19^. Surprisingly, however, no studies have investigated the consequences of early-life conditions for differences in reproductive ageing rates in humans or other similarly long-lived animals. Understanding factors that contribute to between-individual differences in reproductive senescence in long-lived species is of increasing interest and importance, because the age at which women desire children is increasing in modernised society. However, although the physiological factors underpinning reproductive senescence have been relatively well studied, the social and ecological factors that drive between-individual variation in reproductive ageing rates have received very little attention.

Here we use a unique longitudinal (born 1941–2014) dataset of Asian elephants employed in the Myanmar timber industry with life histories approximating wild rather than zoo animals^20^, to determine how variation in early-life conditions shapes reproductive ageing rates. Asian elephants are of interest to ageing studies because their lifespan (max. 80 years) allows comparison with humans, shedding light on why a species with a long reproductive life and high cost of reproduction^21^ might not also evolve menopause^21^. The distant relatedness of elephants to humans means any similarities or differences evolved in convergence. The Myanmar elephant dataset is suitable for investigating these topics for several reasons. First, Asian elephants have extremely long generation times (~18 years) and extended periods of maternal care (with weaning age at about 5 years)^22^,^23^,^24^,^25^. In this regard, they provide a unique data-point at top end of the longevity-care distribution in animals, enhancing our ability to make generalisations regarding the fundamental basis of senescence theory^26^. Second, similarly to elsewhere in Asian elephant range area, in Myanmar, individuals are born in distinct seasons, allowing tests of the effects of birth conditions on patterns of reproductive senescence: cool (November-February); hot (March-June); and monsoon (July-October), with the monsoon in this population being associated with increased maternal work-loads and diseases^27^,^28^,^29^. We have shown that whilst elephants can breed throughout the year, with continuous 16 week reproductive cycles^30^, there is seasonal variation in births. This birth variation is associated with higher than expected conceptions in the annual resting period (beginning in February and ending with the return to work in June)^31^. This could be a result of mothers avoiding reproducing outside the optimal time. Offspring born outside this peak have lower survival to independence at age 5^31^, however, the long-term effects of being born outside of this peak season are not known. Third, our data allow us to investigate the effects of stress on patterns of reproductive senescence and faecal glucocorticoid metabolites have been shown to be an excellent indicator of energetic stress in closely related African elephants (*Loxodonta africana)*^32^. Although we don’t artificially manipulate the environmental stressors as is possible in shorter-lived model species, this study design comes as close as possible to such manipulations, as the workload and climatic conditions vary consistently. Fourthly, there is evidence that early life conditions affect future reproductive potential in African elephants^33^ and poor conditions have immediate effects on demography^34^. Finally, Asian elephants are classified as endangered by the International Union for the Conservation of Nature^35^ and understanding the causes of reproductive scheduling and variation in reproductive success may have potentially significant ramifications for the conservation of this flagship species. In particular, current fertility rates are below replacement level and pose a risk to both captive and wild populations^36^ and therefore any early life interventions to improve female reproductive rate over the subsequent decades could be of large practical importance.

In this study, we first (*i*) measured the stress level variation in reproductive-aged females, we then characterised “high stress” and “low stress” periods using the physiological marker of faecal glucocorticoid metabolites, rather than proxies such as climatic season or exposure to workload. Based upon these newly defined variables marking poor and more amenable birth conditions, we then (*ii*) linked this to differences in reproductive ageing rates of daughters, and (*iii*) finally added all those effects up to quantify consequences on lifetime reproductive performance. We hypothesise that being born at the time of year in which elephants are most stressed could be associated with long-term effects on the reproductive success of females and their pattern of reproductive senescence^3^,^4^. We predict that “high stress” birth season may be associated with reduced likelihood of reproduction, fewer offspring produced or higher investment in reproduction at younger ages, and a more rapid reproductive senescence^11^.

## Results

**Figure 1:**
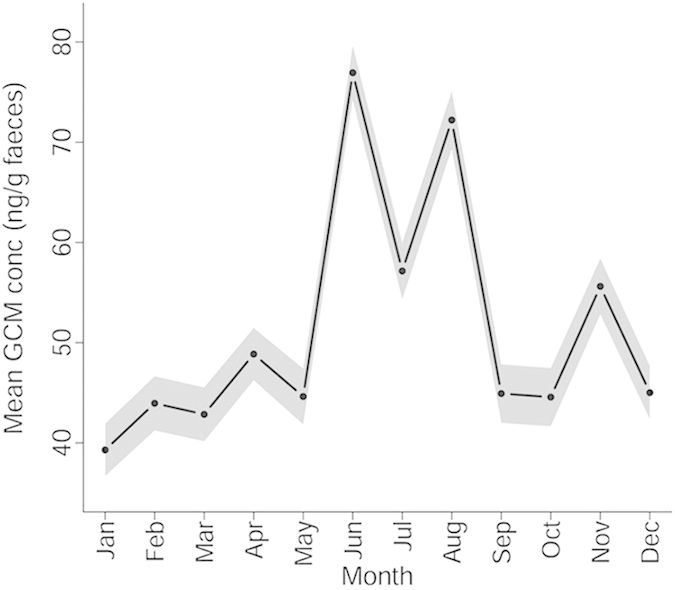
Mean monthly glucocorticoid metabolite concentration in female elephants aged 17-55. We used a total of 354 points for 37 reproductive-aged females. The solid line indicates mean predicted values for monthly glucocorticoid metabolite concentration (GCM). The shaded area indicates standard error around the mean values.

Our analyses reveal first a clear pattern of seasonal variation in maternal stress levels. We analysed glucocorticoid metabolite concentration in non-invasively collected fecal samples from non-pregnant non-lactating reproductive-aged (17–55 years) females (354 samples from 37 females) for a complete annual monsoon cycle (Fig. 1) shown to be highly consistent between years^37^. The sampling year was a typical one in terms of weather conditions^38^. The average stress hormone concentration in the ‘high stress’ months of June, July and August was 46.0% higher compared to the other months, consistent with a larger dataset of both sexes^38^. Using ‘high stress’ season rather than month to predict glucocorticoid metabolites in these females produced a better fitting model (likelihood ratio test for model with ‘high stress season’ vs. month; Χ^2^_1_ = 55.18, P < 0.001). This ‘high stress season’ corresponds to return to work after the annual 3-month rest period and overlaps with the monsoon season (late June-October). These results corroborate our findings showing that birth rates across several decades have consistently been at their lowest during these months and that any resulting calves have lower survival prospects to independence^31^.

**Figure 2:**
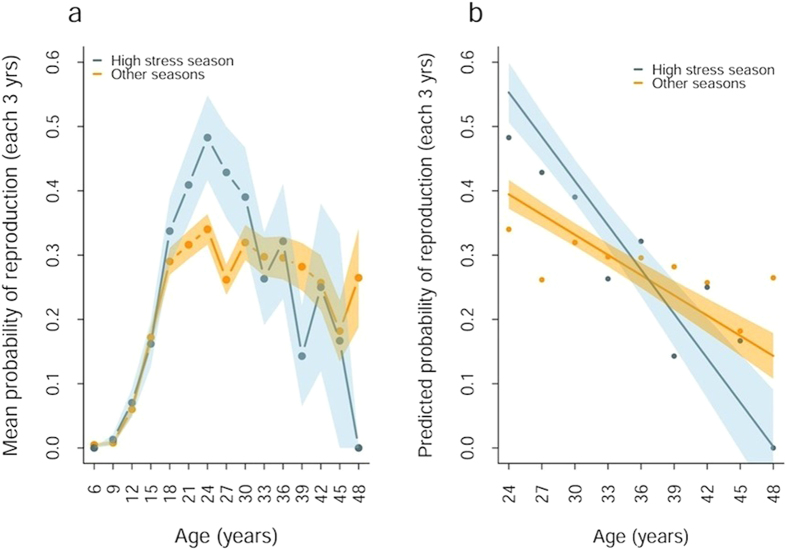
Mean three yearly reproductive rates (a) across the lifespan and (b) in the senescent phase in female Asian elephants by high stress birth season. In (**a**) The raw age-specific means (points) suggest an initial increase, followed by a peak at 24 and subsequent decline (also found in model1524, Table S3). The points show the mean probability of reproduction for all individuals by birth season in the raw data (6,483 records from 1,078 female elephants) and the shaded areas show the standard errors around these points. In (**b**) Mean three yearly probability of reproduction in female Asian elephants aged over 24 by ‘high stress’ birth season (2,059 data points from 455 females). The lines show predictions from the best fitting model (Table 1) fitted to individuals aged over 24, the shaded areas are SE values from the same model. The points show the mean probability of reproduction for each 3-year interval in the raw data by ‘high stress’ birth season.

Second, birth during this ‘high stress’ season has a strong effect on age-related female reproductive success. In general, females had low probabilities of reproducing between 5–16 years, followed by a steep increase to age 24 and a slow decline (Fig. 2a). However, females born in the ‘high stress’ (high workload for working elephants, monsoon conditions and increased glucocorticoid concentration) season exhibit faster reproductive senescence following the peak of reproduction (Fig. 2b; interaction between age and birth season, Χ^2^_(d.f.=1)_ = 6.07, P = 0.014, Table 1A) than females born in lower stress months. The same pattern is observed on the reduced sample with birth order included (interaction between age and birth season, Χ^2^_(d.f.=1)_ = 8.96, P = 0.0028, Table 1B) and the effect of birth order itself is not significant (likelihood ratio test on models with and without birth order term Χ^2^_(d.f.=1)_ = 0.03, P = 0.863). This is also the case for the effect of maternal age (interaction between age and birth season with maternal age in the model, Χ^2^_(d.f.=1)_ = 8.79, P = 0.0030, Table 1C; likelihood ratio test on models with and without maternal age term Χ^2^_(d.f.=1)_ = 0.22, P = 0.636). Furthermore, interactions between birth order and birth season and maternal age and birth season did not improve the fit of models and were therefore discarded (likelihood ratio tests for models with and without the interaction term for birth order and birth season Χ^2^_(d.f.=1)_ = 1.76, P = 0.184 and for the interaction between maternal age and birth season Χ^2^_(d.f.=1)_ = 0.276, P = 0.599). This suggests that the effect of birth season on female reproductive pattern in adulthood is unlikely to be confounded by only certain type of mothers (such as inexperienced or lower ranking females) reproducing at the first place during the high stress season, leading to their daughters showing lower performance in adulthood. Notably, when we repeated these analyses based on “high stress” conception season, there was no significant interaction between conception season and age on reproduction (likelihood ratio test for models with and without interaction between conception season and stress Χ^2^_(d.f.=1)_ = 0.253, P = 0.615 Table S1).

Initially, ‘high stress’-born females have somewhat higher probability of reproducing at the peak age of 24 (mean ± SE = 0.46 ± 0.064) than those born at other times of year (0.34 ± 0.024), but this was not statistically significant (t-test for difference in means = −1.785, p = 0.078). However, due to their faster reproductive senescence, females born in the ‘high stress’ season had 15.9% fewer calves in their lifetime than those born in the rest of the year (effect of birth season Χ^2^_(d.f.=1)_ = 7.98, P = 0.005). This difference does not appear to be attributable to differences in failure to reproduce at all in a lifetime (effect of birth season Χ^2^_(d.f.=1)_ = 1.37, P = 0.24). Rather, it is due to the faster decline in reproduction from the age of 24 among females born in the ‘high stress’ season, with the probability of reproduction declining by 2.2% during each 3-year period in ‘high stress’-born females, compared to only 0.73% in those born in the rest of the year.

## Discussion

Our findings that females born during high-stress months exhibit faster reproductive senescence in adulthood and have significantly reduced lifetime reproductive success than their counterparts born at other times of year have significant implications for our understanding of the later life reproductive consequences of early life conditions, defining conservation strategies in endangered Asian elephants and finally for our understanding of the application of theories of ageing to long-lived species. Poor early life conditions could affect offspring health and physiological response to stress, and consequently alter their reproductive schedule across life. For example, at a cellular level, many types of stress, including DNA damage, oxidative stress, oncogenic stress, epigenetic alterations and telomere dysfunction have been linked to senescence^39^. Interestingly, although the ‘high stress’ months in our study coincide with peak adult body condition and food availability in the monsoon^40^, there are still long-term negative consequences of the ‘high stress’ birth conditions. One potential explanation is that glucocorticoid metabolite concentration peaks in the months of June to August because these are associated with the beginning of the monsoon and the work season in this population, thereby indicating that the environment has strong effects on the demography of this population. Therefore, elephants born during the time of the year when glucocorticoid metabolite concentrations are highest are born when mothers are energetically most challenged (possibly due to increased workload or climatic conditions) and therefore suffer some cost later in life. Even pregnant females could experience these changes because i) pregnant mothers continue to work until they are determined to be pregnant (likely to be at least a year into the 22-month gestation) and ii) all elephant age groups and sexes experience a peak in glucocorticoid metabolites at this time and it may therefore also be linked to other characteristics of the environment than workload^38^. To our knowledge, this is the first study to show that variation in birth conditions affects the decline in age specific reproductive rates in a long-lived mammal and our results contribute a vital data point to the small sample of ageing studies in natural mammalian populations, and allows comparisons with human senescence.

Recent experimental studies on rodent models have focused on determining the windows during development that are most sensitive to environmental disturbances and lead to downstream effects on different outcomes. Such studies have shown, for example, that exposure to maternal stress early in gestation in lab rats has no or little effect on offspring hypersensitivity to glucocorticoids^41^ whereas exposure late in gestation has a large effect on glucose tolerance^42^ and sensitivity to glucocorticoids, whilst hypertension was linked to stress exposure throughout pregnancy^41^. Although we show in elephants strong seasonal variation in glucocorticoid metabolites of potential mothers, we cannot state without a longitudinal study over several decades what specific effect high stress birth conditions is a proxy for, nor whether it is exposure in the mid-pregnancy, around the time of birth or postnatal exposure, which drives the effect. Because of the 22-month gestation of the elephants, each individual experiences up to two “high stress” seasons *in utero*. Therefore the “high stress”-born individuals also experienced such a season in the middle of pregnancy (around month 8–10). Our additional analyses show that individuals conceived in the “high stress” season (and experiencing it again 12 months into gestation) do not experience a difference in the pattern of reproductive senescence similarly as those born during the “high stress” season do. This supports the idea that the window in development in which the effect of “high stress” season occurs may be later in pregnancy, in collaboration of studies e.g. in humans that have reported the largest effect of poor prenatal developmental conditions on outcomes such as glucose tolerance during later rather than earlier stages of pregnancy, although other effects such as coronary heart disease and obesity are associated with early gestation exposure^43^. It should be noted however that reproductive senescence is the a sum of many underlying traits such as foetal ovarian development and number, and other traits that affect longevity and resource allocation that may all respond differently to insults at different developmental windows^44^, and only experimental work on short-lived model species can address these mechanistic questions satisfactorily. Our study shows a correlation between “high stress” birth season and reproductive ageing pattern, which could be operating through many mechanisms, such as growth rate^45^, which may affect the timing of reproduction^46^.

Regardless of the mechanism, our key conclusion is that annually fluctuating conditions linked to time of birth appear to be associated with reproductive rate decades later and are an important source of variability in this population. Such findings are of interest in the light of findings from another long-lived aseasonal breeder, humans, showing significant differences between individuals in longevity^47^ and lifetime reproductive success^48^ depending on their month of birth. Such variation in humans depending on their birth season has not so far been linked to their subsequent reproductive senescence. The variability in our elephant population according to the birth season could arise either from certain females systematically avoid giving birth at this time, for example multiparous mothers are less likely to give birth in these months^31^, or through affecting the offspring of those that do. If the former is the case, managing stress, e.g. through workload, rest time or provisioning in reproductive females is of paramount importance in order to improve management, reproductive rates and conservation of this endangered species. As first-time mothers are more likely to give birth outside the months with highest birth rate, primiparous mothers could be offered additional attention and medical care. However, it should be noted that birth order and maternal age do not appear to affect reproduction in this study and controlling for these does not alter our conclusions. If the effect is therefore on the offspring, in terms of management, particular focus could be placed on early life care for calves born in the high stress season. Under both scenarios, there is the possibility to improve calf survival and potentially future reproductive prospects.

More generally, our results suggest that improving developmental conditions could delay reproductive ageing in a species as long-lived as humans, but with a different pattern of reproductive ageing that appears not to include menopause^21^. This is of interest, because the age at which women desire children is increasing in western societies^49^ and there is currently an increased need to improve reproductive health and ability at advanced ages as well as to identify early those individuals who may be at risk of low late-life fertility. Studies on early life origins of health and disease have focused on designing interventions that might mitigate the negative effects of poor early conditions, such as low birth weight or gestational age, on later life disease outcomes^50^. Variation between women in age at menopause^51^ suggests that there is likely to be variation between women in their rates of reproductive senescence, but the social and ecological factors that drive between-individual variation in ageing rates have not been studied in humans^52^. Our finding that birth during periods when females have highest stress levels in long-lived Asian elephants may be linked to reproductive ageing of their daughters offers interesting insights into examining such variation and its causes also in humans.

Our results are also of interest in the light of recent literature on the potential benefits of poor early life conditions in certain situations. For example, prenatal caloric restriction has been found to be correlated with changes in metabolic phenotype such as altered metabolism of glucose and insulin as well as changes in body composition such as central adiposity and reduced muscle mass^52^. The beneficial later life consequences of such changes when the postnatal environment and prenatal environment are matched is highlighted in certain literature^52^. However in line with wild animals studies^11^,^53^,^54^ and humans^19^ our results actually support the opposite case with poor early conditions having both short term (survival) and long-term (reproductive senescence) costs. Future research on long-term consequences of birth conditions, including objectively measured physiological markers such as glucocorticoid metabolites and environmental markers such as resource abundance, on offspring reproductive senescence should thus be of great interdisciplinary interest.

## Methods

### Demographic data

Asian elephants are recognised as endangered^35^: the remaining populations are distributed discontinuously across Asia with a total wild population of 38,500–52,500, and further *in situ* captive elephants of 16,000^55^. Throughout their range elephants live in tropical monsoon climates characterised by high seasonal variation in temperature, rainfall and food availability^37^. However, whilst the number of births recorded varies throughout the year, elephants can be defined as continuously rather than seasonal breeders^31^. Consequently, offspring in the same population can have very different birth conditions and given the 20–22 month gestation period^30^, each individual experiences almost two complete monsoon cycles *in utero*, with the possibility of high resource availability months falling at different stages of development^31^,^56^. Elephants are also long-lived, with survival up to 60 years being common and maximum known lifespans of >80 years^28^,^57^ and reproductive lives that can span 50 years^21^. These characteristics make Asian elephants an interesting candidate species in which to study the effects of variation in early developmental conditions on reproductive ageing.

Myanmar has the largest remaining population of captive elephants worldwide numbering approximately 5,000 individuals. In Myanmar, elephant draught power has been utilised extensively in timber harvesting for over a century^58^ and currently ~2,700 state-owned captive elephants are employed in the timber industry under the management of Myanma Timber Enterprise (MTE). Our database includes all government-owned working elephants in the country and therefore elephants that are moved within the country remain in the dataset. These elephants are not provisioned and live in forest camps, where they are used during the day as riding, transport and draught animals. After the end of the working day, elephants are released into the forest for the night and are able to forage in their family groups unsupervised and encounter captive and wild conspecifics. The population breeding rates are natural and not managed by humans, with many calves thought to be sired by wild bulls. Calves born in captivity are cared for by their biological mothers and allomothers, and are weaned at age 4–5 years^58^ at which point they are separated from their mothers and tamed. Working females are rested from mid-pregnancy (around 12 months into gestation or when the pregnancy can be diagnosed based on visible changes to the mammary tissue) until their calf is one year old^57^,^58^. They are then given light scheduled work or used as baggage or transport animals for 3–4 hours per day but allowed to nurse the calves on demand.

For our analyses, we utilise a unique and extensive demographic dataset detailing the life histories of the timber elephant population of Myanmar for nearly a century. These data have been compiled using the elephant logbooks and annual extraction reports archived and maintained by the Extraction Department of Myanma Timber Enterprise. The state ownership of thousands of elephants makes it possible to compile and transfer data of all registered elephants from the individual based-logbooks to a database. The data recorded for each individual include: registration number and name; origin (wild-caught or captive-born); date and place of birth if captive-born or place and time of capture if wild-caught; mother’s registration number and name if captive-born; year; dates and identities of all calves born; date of death or last known date alive; and cause of death. The elephants have never been culled, released or sold and are kept through their lives even if they are unable to work or reproduce, ensuring that our sample is not biased towards the most healthy or reproductive individuals. Only basic veterinary care is available (for example, treatment of injuries and limited vaccinations), and the survival of the elephants is close to survival rates observed in wild populations^20^,^59^. All MTE-owned elephants are and have historically been subject to strictly enforced and documented regulations regarding workload; the central government dictates the hours of work per week, working days per year, and tonnage of logs to extract per elephant. For example, in 2010 all mature elephants (aged 17–44 years) worked for 3–5 days a week (depending on weather and forage availability) for an average of 5–6 hours a day (maximum 8 hours) with a break at noon, and the maximum tonnage of logs allowed to be dragged/pulled by each elephant in a year was 400 tons. All of the elephants finish their work season by mid-February each year, which coincides with the hottest and driest time of year, and work resumes around mid-June coinciding with the beginning of the monsoon season. This work schedule has altered little over the past 40 years.

This study uses only records of captive-born mothers and their offspring because precise date and place of birth are known for these individuals and because methods of capture are associated with reduced later fitness in wild-captured elephants^58^. After these restrictions were applied, the sample analysed here consists of 1078 females, born 1941–1999, all of whom survived to at least age 5 years from 10 regions covering the whole of Myanmar (Ayeyarwady, Bago, Chin, Kachin, Magway, Mandalay, Rakhine, Sagaing, Shan and Tanintharyi). Our coverage means no elephants dispersed from the population, and all censored elephants were alive at the end of the study. The youngest female reproducing in the sample was 5 years old and the oldest was 53 years old (mean age at first birth: 19.48; median: 19 years), with the maximum lifetime number of calves being 10. In this population, 26% of all calves born failed to reach their fifth birthday, the age they are removed from their mothers and trained^58^. These life-history patterns mirror data found for wild populations of Asian and African elephants^60^,^61^,^62^. These data were collected in accordance with University of Sheffield guidelines as part of a NERC funded project. As such all experimental protocols were approved by the University of Sheffield ethics committee.

### Hormonal data

In order to determine the high stress months in reproductive-aged female elephants, and to investigate the association between birth in the high stress season and reproductive senescence, we collected longitudinal faecal sample data for within-individual hormone variation analysis (glucocorticoid metabolite concentration). We took samples on a monthly basis for 12 months from 37 female MTE elephants aged 17-55 from December 2011 to November 2012. We used non-pregnant rather than pregnant females to measure the variation in glucocorticoid metabolites experienced by potential mothers, rather than pregnant individuals whose hormone profiles are likely to be affected by pregnancy. Furthermore as cows are only determined to be pregnant using visual and behavioural cues, they could be at different stages of pregnancy when diagnosed, thus preventing any characterisation of the conditions that their foetuses would have experienced around conception. We also tested the variation of these metabolites in other age and sex classes^38^. We extracted glucocorticoid metabolites (see below) from faecal material using a validated protocol for boiling extraction and enzyme immunoassay for glucocorticoid metabolites^63^. We defined high stress as consecutive months that showed significantly higher average glucocorticoid metabolite concentration per gram of dry faeces than the January baseline. The glucocorticoid metabolite concentrations (GMC) vary significantly by month (F for factor month = 32.24, DF = 11, P = <0.0001), and the consecutive months with highest concentration were June, July and August in reproductive aged females. This pattern was also followed in females of other ages and males^38^. The average glucocorticoid metabolite concentration in the ‘high stress’ months of June, July and August was 68.1 ng/g faeces compared to 45.6 ng/g faeces in the other months. This “high stress” season (June-August) substantially overlaps with the monsoon season (late June to October). Using ‘high stress’ season (binary term) rather than month (12 level factor) in GLMMs predicting GMC (continuous) by age group (factor with 3 levels), ID (random term) and birth year (random term) significantly improved the fit of the model (likelihood ratio test for model with ‘high stress season’ vs. month; Χ^2^_1_ = 55.18, P < 0.001). These months will therefore be characterised as the high stress season in further analyses.

### Statistical Analyses

#### Age-related variation in reproduction

First, we characterised curves of age-specific reproductive rate in order to determine when senescence begins. We analysed reproductive senescence by implementing logistic regression models as generalised linear mixed effect models to predict the probability of reproduction (binary; 0 = did not produce a calf, 1 = produced at least one offspring) for each 3-year observation period in an elephants life from the earliest recorded reproductive age (5 years) onwards, sample sizes for each age bin ranged from 1,078 in the earliest (age 5–7 years) to 38 in the last (age 47–49 years) (see Table S2 for a complete list of sample sizes). We chose to use 3-year rather than annual observation periods because the 22-month gestation of elephants and prolonged lactation mean that females are not able to reproduce each year and the average inter-birth interval is 5.4 ± 2.7 years^59^ in this population. In the 3 cases in which elephants reproduced twice in 3 years, their reproduction was scored as 1. The model with all potentially relevant fixed effects included: birth season (modelled as high stress season or not), birth decade (5 categories), censored (binary 0 = not censored, 1 = did not die before the end of the study) and age at last sighting (linear and quadratic terms). Random terms were repeated measurements (individual ID) and location (region). In order to test the possibility that any effect of birth season might be confounded by a different, “lower quality” subset of mothers initially giving birth during the high stress season, we repeated the models on a reduced sample (N = 562) that had data on birth order (taken as a surrogate of maternal experience; a binary term 0 = first-born, 1 = later-born) and maternal age (surrogate of social rank; a continuous term) to control for differences in maternal effects (this subset covered only 3 birth decades).

For all models, we restricted our dataset to individuals for which all variables were available, leading to a total of 6,483 elephant-three-year observations from 1,078 elephants, among which 1,127 reproduction events were recorded. Of these elephants, 832 did not die before the end of the study period (see above for treatment of censoring in the models). We used precisely known ages at last observation to account for differences between censored and dead individuals according to^46^. All analyses were conducted in R (R Core Team. R: A language and environment for statistical computing. *R Foundation for Statistical Computing*, 2013) and GLMMs were implemented using the package *lme4* (Bates D, Maechler M, Bolker B. lme4: Linear mixed-effects models using S4 classes, 2011).

We determined the shape of the age function by comparing candidate GLMMs using AIC. First, we fitted a null model without age terms and models with linear, quadratic and cubic age terms (Table S3). We then compared these to more flexible threshold models with a range of age breaks that characterise the age-related changes in reproduction (Table S4 and S5), following similar methods used to characterise age-related changes in reproductive rate in previous studies^46^. First, we fitted models with a single threshold; the first age bin was 5–7, presented here as the mean age of 6 and we then fitted models with a single threshold break from age 9 (age bin 8–10) to age 45 (age bin 44–46). Second, we fitted models with two thresholds at three-year intervals; with the first between 12 and 39 years and second between 18 and 45 years with a minimum of 3 points within each threshold. This method allowed the linear slope of annual breeding success to vary independently between each threshold and ensured more than one age bin separated each threshold. In all of the above models, the terms age and age at last observation were divided by 100 to aid model convergence, and following this procedure all models converged successfully.

The best-supported final model describing age-related variation in reproduction included two age thresholds (Table S3) at 15 and 24 (i.e. bins 14–16 and 23–25) and these indicate that the reproductive ageing trajectory was comprised of three stages: first, a period of low breeding success at ages 5–15; second, a rapid period of increase between ages 15–24; and finally, decline from age 24 onwards. Next, in order to focus on the effect of birth season on reproductive senescence, we hereafter limited the data to ages after the last threshold of the best fitting model (i.e. age 24 onwards when senescence begins) allowing us to only investigate the intercept and slope in the ageing stage (Table 1A). We then analysed the data using GLMMs to test the effect of age and birth season on probability of reproduction, whilst controlling for confounding factors as above. We repeated analyses using conception season (based on a gestation duration of 22 months).

#### Birth season and lifetime reproductive success

To determine the lifetime fitness consequences of birth season, we investigated the effect of birth season on (a) probability of reproduction throughout life and (b) the total number of calves born to a female in her lifetime. First, we used GLMMs with a binomial error structure with a binary response variable to investigate whether or not a female ever reproduced, where 0 = produced no offspring across life and 1 = produced at least one offspring. Next, we used GLMMs with a Poisson error structure to analyse data on lifetime reproductive success. In each model, we were chiefly interested in the binary explanatory variable of whether the focal females was born in the high stress season (months June to August, 1) or not (0). We controlled for cohort effects (birth decade as a factor with 5 levels), age at last sighting (modelled as a linear and quadratic term), whether an individual was censored or not (a binary variable where 0 = not censored and 1 = censored) and random terms to take into account individual variation (individual ID) and regional variation (region as a factor with 10 levels) in all models. We used the full dataset (n = 1078) in both models (Table 2A and B).

## Additional Information

**How to cite this article**: Mumby, H. S. *et al.* Elephants born in the high stress season have faster reproductive ageing. *Sci. Rep.* **5**, 13946; doi: 10.1038/srep13946 (2015).

## Supplementary Material

Supplementary Information

## Figures and Tables

**Table 1:** Model of effects of birth in high stress season on (A) three-yearly breeding success in individuals aged 24 (age bin 23–25) and over (B) a subset of these individuals with a term for birth order and (C) a subset with a term for maternal age.

Fixed effects	Estimate	Std. Error	z value	Pr(>|z|)
(A) Probability of reproduction over 24				
(Intercept)	−2.122	2.037	−1.042	0.297
‘High stress’ season	2.374	0.839	2.829	0.005
Age at last sighting	16.768	10.463	1.603	0.109
Age at last sight squared	−0.203	0.130	−1.555	0.120
Age	−0.051	0.009	−5.385	<0.001
Censored	0.223	0.154	1.448	0.148
Born in 1960’s	−0.623	0.183	−3.399	0.001
Born in 1970’s	−1.181	0.240	−4.922	<0.001
Born in 1980’s	−1.868	0.378	−4.941	<0.001
‘High stress’ birth season:Age	−0.066	0.027	−2.405	0.016
Random effects	Variance	Std. Dev		
ID	0.192	0.438		
Region	<0.001	0.001		
(B) Subset including birth order				
(Intercept)	0.342	4.242	0.081	0.936
‘High stress’ season	8.754	3.184	2.750	0.006
Later-born	−0.039	0.236	−0.167	0.867
Age at last sighting	6.721	23.812	0.282	0.778
Age at last sighting squared	−0.093	0.319	−0.291	0.771
Age	−0.067	0.030	−2.226	0.026
Censored	0.442	0.453	0.975	0.329
Born in 1970’s	−1.254	0.792	−1.583	0.113
Born in 1980’s	−2.208	0.887	−2.490	0.013
‘High stress’ birth season:Age	−0.310	0.121	−2.565	0.010
Random effects	Variance	Std. Dev		
ID	0.106	0.326		
Region	<0.0001	0.001		
(C) Subset including maternal age				
(Intercept)	0.256	4.186	0.061	0.951
High stress’ season	8.683	3.176	2.734	0.006
Maternal age	0.004	0.009	0.466	0.641
Age at last sighting	6.341	23.474	0.270	0.787
Age at last sighting squared	−0.088	0.315	−0.281	0.779
Age	−0.067	0.030	−2.242	0.025
Censored	0.468	0.458	1.021	0.307
Born in 1970’s	−1.268	0.784	−1.617	0.106
Born in 1980’s	−2.210	0.885	−2.498	0.013
High stress’ birth season:Age	−0.307	0.121	−2.543	0.011
Random effects				
ID	0.113	0.336		
Region	<0.0001	0.001		

Models (A–C) are generalised linear mixed models with a binomial error structure in which reproduced or not each 3 years was fitted as the binary response term. Parameter estimates are on a logit scale. The cut off of age 24 is based on model 1524 that indicates age specific reproductive rates in the population decline after this age bin 24 (representing the binned ages 23–25). In (A) the analysis was performed on 2,059 records from 455 female elephants that survived to age 24 (no elephants born in the 1950’s survived to age 24 and none born in the 1990’s reached 24 before the end of the study). In (B,C) the analysis was repeated on subset of 562 individuals with data on birth order (binary term: first or later born).

**Table 2:** Model of effects of birth in high stress season on (A) total number of calves produced throughout life and (B) probability of reproduction throughout life in Asian elephants.

Fixed effects	Estimate	Std.Error	z value	Pr(>|z|)
(A) Total number of calves produced				
(Intercept)	−3.311	0.420	−7.884	<0.001
‘High stress’ birth season	0.252	0.086	2.921	0.003
Age at last sighting	0.210	0.022	9.426	<0.001
Age at last sighting squared	−0.002	0.000	−7.372	0.000
Censored	−0.009	0.082	−0.107	0.915
Born in 1950’s	−0.398	0.097	−4.119	<0.001
Born in 1960’s	−0.699	0.127	−5.487	<0.001
Born in 1970’s	−1.001	0.172	−5.808	<0.001
Born in 1980’s	−3.016	0.410	−7.359	<0.001
Born in 1990’s	−3.397	1.035	−3.283	0.001
Random effects	Variance	Std. Dev		
ID	0.014	0.118		
Region	0.019	0.138		
(B) Lifetime probability of reproduction				
(Intercept)	1.396	13.282	0.105	0.916
‘High stress’ birth season	0.418	0.344	1.215	0.224
Age at last sighting	0.368	0.099	3.696	<0.001
Age at last sighting squared	−0.004	0.002	−2.724	0.006
Censored	0.164	0.359	0.458	0.647
Born in 1950’s	−6.850	13.219	−0.518	0.604
Born in 1960’s	−7.718	13.223	−0.584	0.559
Born in 1970’s	−8.369	13.230	−0.633	0.527
Born in 1980’s	−10.433	13.243	−0.788	0.431
Born in 1990’s	−10.131	13.271	−0.763	0.445
Random effects	Variance	Std. Dev		
ID	1.098	1.048		
Region	0.041	0.201		

Model (A) has a poisson error structure in which the number of births was fitted as a count response term. Model (B) is generalised linear mixed model with a binomial error structure in which reproduced or not in the whole life was fitted as the binary response. Parameter estimates for (B) are on a logit scale. For both models, the analysis is based on 1,078 female elephants.
