# Supplementary material for: Elephants born in the high stress season have faster reproductive ageing
Source: Sci Rep. 2015 Sep 14;5:13946. doi: 10.1038/srep13946 (PMC4568471; doi:10.1038/srep13946)
Supplement: Supplementary Information [file srep13946-s1.doc]

## Elephants born in the high stress season have faster reproductive ageing

Hannah S. Mumby^1^*, Khyne U. Mar^1^, Adam D. Hayward^1,2^, Win Htut^3^, Ye Htut-Aung^4^, Virpi Lummaa^1^

^1^Department of Animal and Plant Sciences, University of Sheffield, Western Bank, Sheffield S10 2TN, United Kingdom

^2^ Institute of Evolutionary Biology, University of Edinburgh, Edinburgh EH9 3JT, United Kingdom

^3^ Ministry of Environmental Conservation and Forestry, Myanma Timber Enterprise, Yangon, Myanmar.

^4^ Department of Veterinary Medicine, Yezin University, Myanmar.

### *Contact: [**h.mumby@sheffield.ac.uk**](mailto:h.mumby@sheffield.ac.uk)

### Supplementary Information

### Supplementary hormonal methods

We collected samples as soon as possible after defecation and stored them in ziplock bags at -20°C until drying in a hot air oven at 50°C. Dry feces were separated from plant material by gentle separation and hormones were extracted from fecal material using a validated protocol for Asian elephants^1^. Each 0.10 g sample of dried fecal material was combined with 5 ml 90% ethanol, and boiled in a water bath for 20 minutes, adding ethanol to keep the volume constant. We then centrifuged the samples for 20 min at 2500 rpm and decanted the supernatants into another new set of labeled tubes. We added 5 ml of 90% ethanol to the fecal pellets and vortexed each sample for 30 seconds. Samples were centrifuged for a further 20 minutes at 2500 rpm and then added to the previously poured off supernatant. We dried down the supernatant and diluted to 1:5 in dilution buffer and analyzed hormonal metabolites by a competitive enzyme immunoassay (EIA) protocol^2^ that was validated for fecal cortisol metabolites in Asian elephants by Watson et al.^1^. The polyclonal CJM006 antibody crossreacts with: corticosterone 100%, desoxycorticosterone 14.25%, progesterone 2.65%, tetrahydrocorticosterone 0.90%, testosterone 0.64%, cortisol 0.23%, prednisolone 0.07%, 11-desoxycortisol 0.03%, prednisone <0.01%, cortisone <0.01% and estradiol <0.01%. Horseradish peroxidase (Sigma–Aldrich, St. Louis, MO; Cat# P8375) and was coupled to corticosterone-CMO (Sigma–Aldrich, St. Louis, MO) using the mixed anhydride method described by Munro and Stabenfeldt^2^.

We diluted the antibody in coating buffer (1:20,000; 0.05 M NaHCO3, pH 9.6), added 50 μl to each well of a 96-well Nunc-Immuno MaxiSorp® microtitre plate (Thermo-Fisher Scientific, UK), covered each plate with a microplate sealer and incubated them overnight at 4°C. We washed the plates five times (0.15 M NaCl, 0.05% Tween 20), and loaded each one with 50 μl/well of corticosterone standard (3.9 - 1000 pg/well; C2505 Sigma–Aldrich, St. Louis, MO) in EIA buffer (0.1 M NaPO4, 0.149 M NaCl, 0.1% bovine serum albumin, pH 7.0) or elephant fecal extracts (diluted 1:70 in EIA buffer) immediately followed by 50 μl/well of horseradish peroxidase conjugate (diluted at 1:20,000 in EIA buffer). Following incubation in dark conditions for 2 h at room temperature (RT), plates were washed five times and incubated with 100 μl/well of 4°C substrate [0.4 mM 2,2′-azino-di-(3-ethylbenzthiazoline sulfonic acid) diammonium salt (ABTS), 1.6 mM H2O2, 0.05 M citrate, pH 4.0), in dark conditions, until average optical density reached 0.8 to 1.0. The resulting optical density of all individual wells was then measured at 405 nm. Sensitivity of the assay was 0.03 ng/ml. The inter-assay coefficient of variation (CV) for the high concentration control was 15.1% and for the low control was 9.4%. The corticosterone EIA was validated for Myanmar elephant feces by demonstrating parallelism between serially diluted samples and the respective standard curve, and 90% recovery of added standard hormone to pooled samples^3^.

**Table S1.** Model of effects of conception in high stress season on three-yearly breeding success in individuals aged 24 (age bin 23-25) and over. The model is a generalised linear mixed model with a binomial error structure in which reproduced or not each 3 years was fitted as the binary response term. Parameter estimates are on a logit scale. The cut off of age 24 is based on model 1524 that indicates age specific reproductive rates in the population decline after this age bin 24 (representing the binned ages 23-25). In the analysis was performed on 2,059 records from 455 female elephants that survived to age 24 (no elephants born in the 1950’s survived to age 24 and none born in the 1990’s reached 24 before the end of the study).

|  | Estimate | Std. Error | z value | Pr(>|z|) |
| --- | --- | --- | --- | --- |
| (Intercept) | -1.934 | 2.102 | -0.920 | 0.357 |
| ‘High stress' conception season | -0.060 | 0.678 | -0.088 | 0.930 |
| Age at last sighting | 17.824 | 10.794 | 1.651 | 0.099 |
| Age at last sighting squared | -0.223 | 0.134 | -1.659 | 0.097 |
| Age | -0.056 | 0.010 | -5.594 | <0.001 |
| Censored | 0.229 | 0.154 | 1.487 | 0.137 |
| Born in 1960's | -0.616 | 0.184 | -3.351 | 0.001 |
| Born in 1970's | -1.204 | 0.241 | -5.002 | <0.001 |
| Born in 1980's | -1.925 | 0.376 | -5.115 | <0.001 |
| ‘High stress' conception season:Age | -0.011 | 0.022 | -0.503 | 0.615 |
| Random effects |  |  |  |  |
| ID | 0.170 | 0.413 |  |  |
| Region | <0.0001 | 0.001 |  |  |

**Table S2 Number of elephants in each 3- year age bin by birth season.** Sample sizes fall each 3-years due to loss of elephants through mortality and censoring.

| Age group | Number of elephants |
| --- | --- |
| 5-7 | 1078 |
| 8-10 | 907 |
| 11-13 | 773 |
| 14-16 | 674 |
| 17-19 | 564 |
| 20-22 | 462 |
| 23-25 | 455 |
| 25-28 | 400 |
| 29-31 | 337 |
| 32-34 | 275 |
| 35-37 | 218 |
| 38-40 | 174 |
| 41-43 | 120 |
| 44-46 | 75 |
| 47-49 | 38 |

**Table S3. Comparison of AIC values of models analyzing ageing-related variation in three yearly breeding success in females aged 5-49.** All models were generalized linear mixed-effects models (GLMMs) with binomial error structures. Models were compared using AIC values, where the best-supported model has the lowest AIC value and is shown in ***bold italics***. ΔAIC, χ², and p values from likelihood ratio tests testing fit of the model in comparison to the best-supported model (which is model 1524). Only the best one- and two-threshold models are shown. Birth season in these models is coded as high stress season vs. outside of high stress season. Analysis was performed on 6,483 records from 1,078 female elephants. Threshold one at 15 represents the bin 14-16 years and threshold 2 at 24 represents the bin 23-25 years.

| **(Model) Structure** | **AIC** | **ΔAIC** | **LogLik** | **Df** | **χ²** | **p** |
| --- | --- | --- | --- | --- | --- | --- |
| (0) BASE | 5393.6 | 655.5 | -2684.8 | 12 | 669.42 | <0.001 |
| (1) Age | 5215.2 | 477.1 | -2593.6 | 14 | 487.04 | <0.001 |
| (2) Age² | 4841.3 | 103.2 | -2405.7 | 15 | 111.16 | <0.001 |
| (3) Age³ | 4893.9 | 155.8 | -2431.9 | 15 | 163.7 | <0.001 |
| (15) Threshold = 15 | 4740.7 | 3.7 | -2352.3 | 18 | 10.64 | 0.031 |
| ***(1524) Threshold = 15 + 24*** | ***4738.1*** | 0 | ***-2347*** | ***22*** |  |  |

**Table S4.** A comparison of single-threshold models analysing ageing-related variation in three yearly breeding success by birth season (high stress season vs. not) in females aged 5-49. All models were generalised linear mixed-effects models (GLMMs) with binomial errors and logit link function. Models were compared using AIC values, where the best-supported model has the lowest AIC value (highlighted in ***bold italics***). The model with best fit is the best-fitting one-threshold model in Table 1 of the main text. ΔAIC values are shown relative to the best-supported model. Analysis was performed on 6,483 elephant-three yearly observations from 1,078 female elephants.

| Threshold Age | Model AIC |
| --- | --- |
| 9 | 4910.97 |
| 12 | 4787.18 |
| ***15*** | ***4740.71*** |
| 18 | 4747.00 |
| 21 | 4789.93 |
| 24 | 4835.77 |
| 27 | 4932.97 |
| 30 | 4986.70 |
| 33 | 5053.05 |
| 36 | 5097.28 |
| 39 | 5145.74 |

**Table S5.** A comparison of two-threshold models analysing ageing-related variation in three yearly breeding success by birth season (high stress season vs. not) in females aged 5-49. “T1” indicates where the first threshold ended and “T2” indicates where the second threshold ended. All models were generalised linear mixed-effects models (GLMMs) with binomial errors and logit link function. Models were compared using AIC values, where the best-supported model has the lowest AIC value (highlighted in ***bold italics***). This model is shown as the best-fitting two-threshold model in Table 1 of the main text. Analysis was performed on 6,483 elephant-three yearly observations from 1,078 female elephants.

|  | T1 |  |  |  |  |  |  |  |  |  |
| --- | --- | --- | --- | --- | --- | --- | --- | --- | --- | --- |
| T2 | 9 | 12 | ***15*** | 18 | 21 | 24 | 27 | 30 | 33 | 36 |
| 15 | 4743.32 |  |  |  |  |  |  |  |  |  |
| 18 | 4740.71 | 4740.27 |  |  |  |  |  |  |  |  |
| 21 | 4756.26 | 4742.33 | 4739.37 |  |  |  |  |  |  |  |
| ***24*** | 4770.87 | 4743.90 | ***4738.07*** | 4750.44 |  |  |  |  |  |  |
| 27 | 4814.87 | 4760.88 | 4741.07 | 4750.85 | 4792.14 |  |  |  |  |  |
| 30 | 4832.04 | 4764.93 | 4741.89 | 4752.51 | 4797.06 | 4839.13 |  |  |  |  |
| 33 | 4857.93 | 4774.62 | 4744.29 | 4753.34 | 4796.70 | 4840.22 | 4939.89 |  |  |  |
| 36 | 4871.23 | 4777.13 | 4743.58 | 4752.69 | 4796.74 | 4840.63 | 4940.30 | 4993.06 |  |  |
| 39 | 4889.53 | 4784.16 | 4745.51 | 4753.31 | 4796.48 | 4840.74 | 4939.96 | 4993.72 | 5059.24 |  |
| 42 | 4899.27 | 4785.96 | 4744.43 | 4751.91 | 4795.29 | 4840.26 | 4938.83 | 4992.40 | 5059.03 | 5102.50 |

**Supplemental References**

1 Watson, R. *et al.* Development of a versatile enzyme immunoassay for non-invasive assessment of glucocorticoid metabolites in a diversity of taxonomic species. *Gen. Comp. Endocrinol.* **186**, 16-24 (2013).

2 Munro, C. & Stabenfeldt, G. Development of a microtitre plate enzyme-immunoassay for the determination of progesterone. *J. Endocrinol.* **101**, 41-49 (1984).

3 Brown, J. L. *et al.* Successful artificial insemination of an asian elephant at the national zoological park. *Zoo Biol.* **23**, 45-63 (2004).
